# Evaluation of a simple tool to assess the results of Ponseti treatment for use by clubfoot therapists: a diagnostic accuracy study

**DOI:** 10.1186/s13047-019-0323-4

**Published:** 2019-03-04

**Authors:** Tracey Smythe, Debra Mudariki, Maxman Gova, Allen Foster, Christopher Lavy

**Affiliations:** 1 International Centre for Evidence in Disability, London School of Hygiene & Tropical Medicine, Keppel Street, London, WC1E7HT UK; 2 University of Witwatersrand, 1 Jan Smuts Avenue, Braamfontein, Johannesburg, 2000 South Africa; 3 Department of Surgery, Parirenyatwa Hospital & University of Zimbabwe, Harare, Zimbabwe; 4 Nuffield Department of Orthopaedics Rheumatology and Musculoskeletal Science, University of Oxford, Windmill Road, Headington, Oxford, OX3 7HE UK

**Keywords:** Clubfoot, Congenital talipes equinovarus, Diagnostic study, Measure, Success, Ponseti, Low income setting

## Abstract

**Background:**

We aimed to develop and evaluate a tool for clubfoot therapists in low resource settings to assess the results of Ponseti treatment of congenital talipes equinovarus, or clubfoot, in children of walking age.

**Method:**

A literature review and a Delphi process based on the opinions of 35 Ponseti trainers in Africa were used to develop the Assessing Clubfoot Treatment (ACT) tool and score. We followed up children with clubfoot from a cohort treated between 2011 and 2013, in 2017. A full clinical assessment was conducted to decide if treatment was successful or if further treatment was required. The ACT score was then calculated for each child. Inter-observer variation for the ACT tool was assessed. Sensitivity, specificity, positive and negative predictive values were calculated for the ACT score compared to full clinical assessment (gold standard). Predictors of a successful outcome were explored.

**Results:**

The follow up rate was 31.2% (68 children). The ACT tool consisted of 4 questions; each scored from 0 to 3, giving a total from 0 to 12 where 12 is the ideal result. The 4 questions included one physical assessment and three parent reported outcome measures. It took 5 min to administer and had excellent inter-observer agreement.

An ACT score of 8 or less demonstrated 79% sensitivity and 100% specificity in identifying children that required further intervention, with a positive predictive value of 100% and negative predictive value of 90%. Children who completed two or more years of bracing were four times more likely to achieve an ACT score of 9 or more compared to those who did not (OR: 4.08, 95% CI: 1.31–12.65, *p* = 0.02).

**Conclusions:**

The ACT tool is simple to administer, had excellent observer agreement, and good sensitivity and specificity in identifying children who need further intervention. The score can be used to identify those children who definitely need referral and further treatment (score 8 or less) and those with a definite successful outcome (score 11 or more), however further discrimination is needed to decide how to manage children with a borderline ACT score of 9 or 10.

**Level of evidence:**

Level II, Diagnostic Study.

**Electronic supplementary material:**

The online version of this article (10.1186/s13047-019-0323-4) contains supplementary material, which is available to authorized users.

## Background

Clubfoot, or congenital talipes equinovarus, is a common deformity where the affected foot is fixed downward and inward. The birth prevalence of clubfoot is estimated in the range of 0.5 to 2.0 cases/1000 live births in low and middle income countries [1]. Most cases of clubfoot occur as an isolated birth defect and are known as ‘idiopathic’ because the cause is not known. The remaining 20% of cases are associated with other structural conditions such as arthrogryposis, syndromes and disorders of the nervous system, for example spina bifida [2]. Male sex is consistently associated with an increased risk of clubfoot; clubfoot affects twice as many boys as girls [3].

There is a global trend toward use of the minimally invasive Ponseti method [4] for the correction of clubfoot, which consists of simultaneous correction of the components of the clubfoot deformity with manipulation and casting. A percutaneous tenotomy of the Achilles tendon is usually required to correct the residual equinus. A foot abduction brace is then needed to maintain the corrected position until 4 years of age [5]; the clubfoot deformity has a strong tendency to recur after corrective treatment because the factors that initiate the deformity remain active as the child grows [6]. Recurrent elements of the deformity are therefore less common after the child is four years old as growth of the foot decreases in speed.

In low resource settings non-specialist health workers are trained as clubfoot therapists [7]. They assess, diagnose, treat and follow up patients with clubfoot [8]. Several scoring systems have been described for clubfoot; these include the Ponseti-Laaveg classification [9] and the Dimeglio classification [10], which are not validated to identify children with recurrent clubfoot who require intervention. The Pirani score [11] is frequently used to assess success during the corrective phase of treatment, however it is not validated for use in children of walking age. The Roye tool [12] measures patient based outcomes in a high-income setting and the Bangla tool [13] was developed to evaluate results in Bangladesh and requires mathematical calculations. There remains no consensus on when intervention of recurrence should occur and elements of the deformity that recur are typically noted under clinical examination and observation of function.

There remains a need for a valid, repeatable and easy to administer tool that will allow clubfoot therapists to differentiate a good outcome of treatment from a less acceptable outcome that needs further intervention. In addition, a standardised method to assess parent reported outcomes after clubfoot treatment is required. To address this gap, we aimed to develop a user friendly, comprehensive tool to assess children of walking age who have undergone Ponseti treatment for clubfoot.

## Methods

This study was conducted and reported according to established STARD (Standards for Reporting of Diagnostic Accuracy Studies) guidelines [14]. (Additional file 1).

### Defining the ACT tool

The Assessing Clubfoot Treatment (ACT) tool was developed through a Delphi process with 35 Ponseti method trainers in Africa. The Delphi study method, criteria, description of consensus and analysis are published elsewhere in detail [15]. The most important criteria for successful clubfoot correction were determined and found to be (i) a plantigrade foot, (ii) the ability to wear a normal shoe, (iii) no pain, and (iv) the parent is satisfied. A literature review was used to develop four possible answers for each of the four identified criteria, and a score given for each answer. The assessment tool was then pilot tested including contextual relevance.

The inter-observer variation for the ACT tool was assessed with two experienced physiotherapists who train and mentor clubfoot therapists in Zimbabwe, and are experienced in co-ordinating national clubfoot programmes. The interclass correlation coefficient was calculated for agreement. The conventional interpretation was used: ≤0.40, poor consistency; 0.41 to 0.74, acceptable consistency; and ≥ 0.75 good consistency [16].

### Study population

A cohort study was established of 218 children with idiopathic clubfoot managed at Parirenyatwa Hospital, Harare. The results of manipulation and casting are published elsewhere [17]. The cohort included all children with a diagnosis of idiopathic clubfoot corrected by the Ponseti method at the study hospital between March 2011 and April 2013 (25 months). The only exclusion criterion was conditions other than idiopathic clubfoot, for example clubfoot associated with a syndrome or comorbidity, e.g. spina bifida.

### Cohort follow up

In January 2017, when patients were 3.5–5.0 years from initial casting, we attempted to follow up all children in the cohort. Phone numbers were extracted from clinic records and carers and their children were invited to participate in the study. Contact was attempted at least three times.

### Study design

First, the ACT tool was administered independently by the two physiotherapists who were experienced in the management of clubfoot in countries in Africa (examiners). Then within an hour a full clinical assessment was performed independently by the two examiners, which involved observation, physical assessment and functional performance review; this included assessment of passive and active range of motion (plantiflexion, dorsiflexion, eversion, inversion of the foot, and knee extension), muscle strength tests of the calf and evertors of the foot), heel raises, squatting ability and gait analysis (walking and running), and discussion with the carer of the child. This examination protocol led to a decision that referral of the child for further treatment (re-casting or surgical review) was required, or that no further intervention was needed. The two examiners then discussed their independent decisions and came to one joint management decision. The examiners were therefore not blind to the decision outcome. The usual process is a clinical assessment by either one or two physiotherapists. The joint management decision was chosen as the gold standard with which to compare the ACT tool. After the decision was recorded, the ACT score was calculated.

### Data collection

The question about the plantigrade position of the foot was answered first by independent physical examination of the child in supine by the physiotherapists, with the knee extended and though the measurement of passive range of dorsiflexion of the hindfoot. The remaining three questions of the ACT score were answered by the carers about the child’s pain, ability to wear shoes and satisfaction. The child followed verbal instructions to complete the functional performance review. In addition, data were collected using a self-administered healthcare satisfaction questionnaire [18] and a quality of life questionnaire [19]. The questionnaires were available in English and Shona and were cognitively tested. Each measure was recorded by hand on a separate paper. The study protocol was pilot tested for suitability before use.

### Data management and analysis

All data were entered into a Microsoft Excel 2000 (Microsoft Inc., Redmond, Washington) software package. Data were analysed using Stata 14.1 (Stata-Corp 4905, Lakeway Drive College Station, Texas 77,845,USA.).

A descriptive analysis compared characteristics of the children who attended follow up with that of the whole cohort.

A comparative analysis of outcomes was explored between three groups of children (a) those who had not completed casting, (b) those who had completed casting and had < 2 years of bracing, and (c) those who completed casting and had 2+ years of bracing.

Sensitivity, specificity, positive and negative predictive values were calculated for the ACT score compared to the gold standard (good outcome or needs referral for further orthopaedic management). A Receiver Operating Characteristic (ROC) curve for the ACT score was created to demonstrate the trade-off between sensitivity and specificity [20].

The potential predictors of the ACT tool were explored. Proportions were calculated for the parent reported outcome measures of healthcare satisfaction and quality of life.

### Ethics, consent and permissions

The Medical Research Council of Zimbabwe (MRCZ/B/789) and the London School of Hygiene & Tropical Medicine (LSHTM ref.:11132) granted ethical approval. The caregiver provided informed written consent. Transport costs were reimbursed.

## Results

The ACT tool consisted of one question about the plantigrade position of the foot answered by physical examination and three questions answered by the carers about the child’s pain, ability to wear shoes and carer satisfaction [15] (Table 1). There were 4 possible answers to each question with a corresponding score from 0 (severe problems) to 3 (no problems). The total score was calculated within a range of 0 to 12.

**Table 1:** ACT questions and score

Score	1.The foot is plantigrade	2.Does your child complain of pain in their affected foot?	3.Can your child wear shoes of your/their choice?	4.How satisfied are you with your child’s foot?
0	Does not reach plantigrade, with additional adduction, cavus or varus	Yes and it often limits their activity	Never	Very dissatisfied
1	Does not reach plantigrade, no additional deformity	Yes and it sometimes limits their activity	Sometimes	Somewhat dissatisfied
2	Plantigrade achieved	Yes but it does not limit their activity	Usually	Somewhat satisfied
3	More than plantigrade i.e. some dorsiflexion	No	Always	Very satisfied

### Inter-observer agreement

The interclass correlation coefficient was 0.99 for questions 1 and 3 and 1.0 for questions 2 and 4.

### Cohort follow-up

Sixty-eight children of the cohort of 218 children (31.2%) attended for assessment in early 2017. The follow up group is representative of the whole cohort in terms of sex, laterality, mean Pirani score at baseline, average number of casts applied, and tenotomy proportion (Table 2). They attended treatment for longer than those not seen, indicating increased compliance with treatment.

**Table 2:** Demographic details of cohort

	Total	Followed up	Not followed up	*P*-value
	N (%)	N (%)	N (%)	
Total	218 (100%)	68 (31%)	150 (69%)	
Sex				
Male	148 (68%)	50 (73%)	98 (65%)	0.23
Female	70 (32%)	18 (27%)	52 (35%)	
Laterality				
Bilateral	119 (54%)	35 (51%)	84 (56%)	0.53
Unilateral	99 (45%)	33 (49%)	66 (44%)	
Tenotomy**:				
Yes	158 (73%)	52 (76%)	106 (72%)	0.43
No	57 (27%)	16 (24%)	41 (28%)	
	Total(95% CI)	Followed up (95% CI)	Not followed up (95% CI)	*P*-value
Average age at first cast	14 months (12–17)	17 months (11–23)	13 months (11–15)	0.16
Mean initial Pirani score				
L foot	3.8 (3.6–4.0)	3.7 (3.4–4.0)	3.8 (3.6–4.0)	0.21
R foot	3.7 (3.5–3.9)	4.0 (3.6–4.3)	3.6 (3.3–3.8)	0.56
Average number of casts	7.2 (6.6–7.9)	6.9 (5.9–8.0)	7.4 (6.6–8.2)	0.44
Average months attending since first appointment	23 months (20–25)	30 months (26–35)	19 months (17–22)	0.0001

**missing data from 3 children

**Fig. 1:**
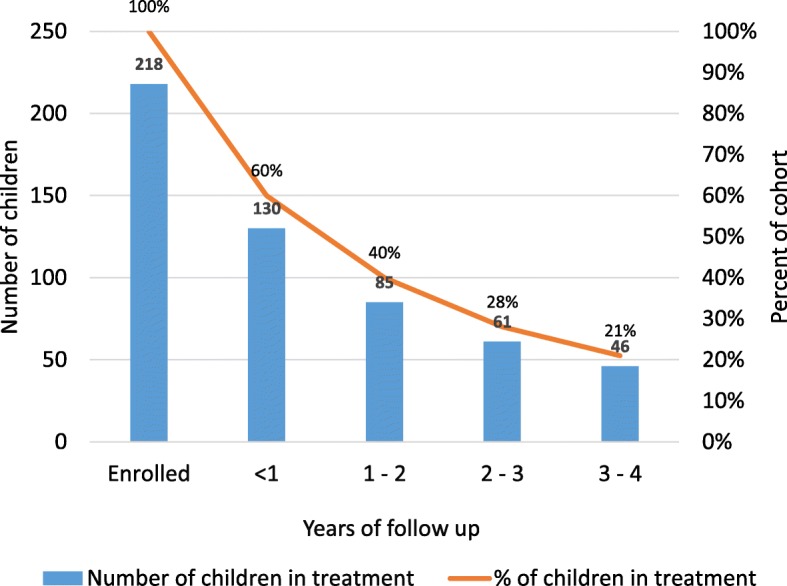
Children attending clubfoot clinic (bracing and correction phase)

By the end of the second year of treatment, 60% of the cohort had stopped attending clinic appointments (Fig. 1).

Of the 68 children seen at follow up, 63 (93%) completed casting and were fitted with a brace, and 38 (56%) used a brace for more than 2 years.

### Clinical assessment

All children were assessed with the reference standard and after the full clinical assessment by the two examiners 44/68 (65%) children were judged not to require any further intervention and 24/68 (35%) were judged to require further treatment (re-casting or surgical review) (Additional file 2). Where there was initial disagreement, consensus on the decision was reached through discussion. No adverse events occurred as a result of any of the outcome measures undertaken.

For the 38 children who finished casting and completed 2+ years of bracing 82% were judged to have a successful outcome (Table 3). Completion of casting and at least 2 years bracing was strongly associated with a successful outcome.

**Table 3:** Outcome at follow-up as judged independently by two expert physiotherapists

	Totals		Finished casting and 2 + years bracing		Finished casting and < 2 yrs. bracing		Did not finish casting	
Recruited at baseline**	218**	100%	83	38%	107	49%	28	13%
Seen at follow-up	68	31%	38	56%	24	35%	6	9%
No intervention required	44	65%	31	82%	12	50%	1	17%
Referral for further orthopaedic intervention	24	35%	7	18%	12	50%	5	83%
*P* value for difference in proportion of those requiring intervention and those not*				0.001		0.06		0.02

**missing data from 8 children (4%) *Fischers exact test

### ACT tool

**Fig. 2:**
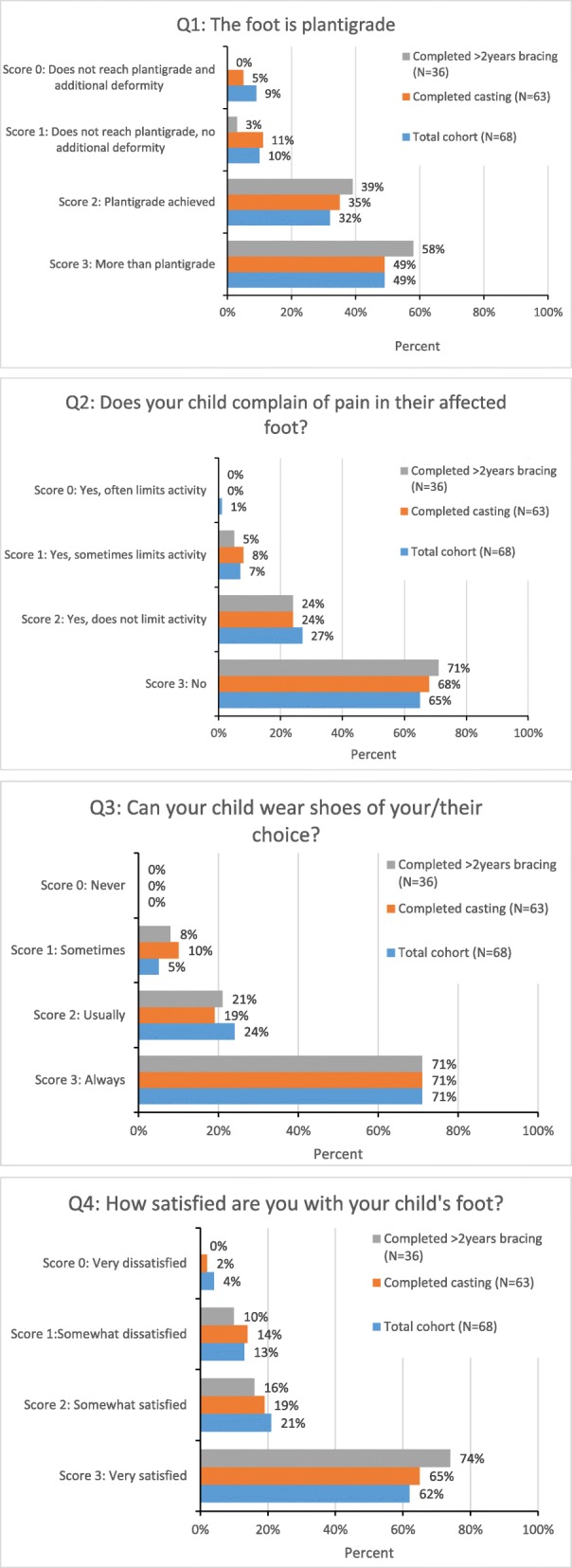
ACT score distribution

55/68 (81%) children achieved plantigrade, and in those who had completed 2+ years of bracing, this increased to 97% (37/38). Scores for parent reported outcome measures increased for children who had completed two years of bracing (Fig. 2). The numerical data are provided in Additional file 3.

72% (49/68) of the children followed up achieved a score of 9 or more. This proportion increased to 84% (32/38) in those who had completed 2+ years of bracing (Table 4).

**Table 4:** ACT score according to compliance with Ponseti treatment

ACT score	Total N	Score ≤ 6	Score 7–8	Score 9–10	Score 11–12
ACT score for total cohort	68	10 (15%)	9 (13%)	13 (19%)	36 (53%)
ACT score for those completing casting	63	8 (13%)	7 (11%)	13 (21%)	35 (55%)
ACT score for those completing casting and bracing for ≥2 years	38	2 (5%)	4 (11%)	9 (24%)	23 (60%)

### Sensitivity and specificity

Given the small sample, 24 children required further intervention of which 19 scored 8 or less on the ACT score (sensitivity: 79%) and the remaining children scored 9 or 10.

Of the 44 children who did not require further intervention on full clinical assessment, all scored 9 or more (specificity: 100%).

A score of 9 or more was found in 49 children, of which 44 were identified as not requiring further intervention (negative predictive value: 90%). Among the children who scored 8 or less, all 19 had been clinically assessed as requiring further intervention (positive predictive value: 100%).

If a score of 9 was used to predict the need for intervention instead of 8, the sensitivity increased from 79 to 83%, the specificity decreased from 100 to 87% and negative and positive predictive values from 90 to 80% and 100 to 91% respectively.

An ACT score of 8 or less correlated with the need for intervention and scores of 11 and 12 correlated with no need for further intervention. An ACT score of 9 or 10 warrants further review.

**Fig. 3:**
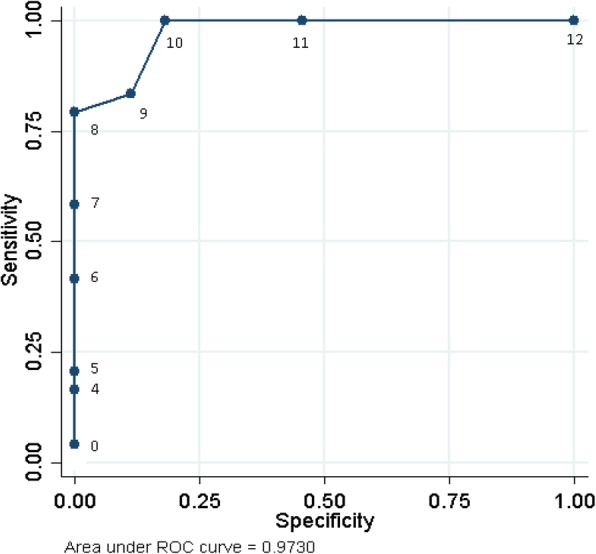
ROC display for ACT score

The ACT score ROC area was 0.97 (95% CI 0.94–1.00) (Fig. 3). The closer the curve follows the left-hand border and then the top border of the ROC space, the more accurate the test.

### Factors affecting ACT score

Children who completed casting and were fitted with a brace were twelve times more likely (95% CI: 1.33–123.49 *p* = 0.03) to achieve a good outcome (score 9–12) than those who did not. Those with 2+ years of brace wear were four times (95% CI:1.31–12.65 *p* = 0.02) more likely to achieve a score of 9–12 than those who used a brace for < 2 years.

Sex, side of clubfoot, age at first clinic attendance, initial severity, number of casts, and tenotomy performed were not associated with an ACT score that predicted need for further intervention (score 0–8) (Table 5).

**Table 5:** Predictors of ACT score

Factor		Success / Borderline (ACT score 9–12) N (%)	Failure (ACT score 0–8) N (%)	Crude OR (95% CI)	*P*-value
Total		54 (79%)	14 (21%)		
Sex	Male	35 (71%)	14 (29%)	1.00	0.85
	Female	14 (74%)	5 (26%)	1.12 (0.34–3.70)	
Clubfoot	Bilateral	24 (69%)	11 (31%)	1.00	0.51
	Unilateral	25 (76%)	8 (24%)	1.43 (0.49–4.17)	
Age first attended clinic	< 2 years	40 (74%)	14 (26%)	1.00	0.42
	≥ 2 years	9 (64%)	5 (36%)	0.57 (0.15–2.19)	
Initial severity (Pirani score)	< 3	34 (69%)	15 (31%)	1.00	0.43
	≥ 3	15 (79%	4 (21%)	1.65 (0.47–5.82)	
Number of casts	≥ 6	28 (68%)	13 (31%)	1.00	0.85
	< 6	21 (78%)	6 (22%)	1.63 (0.53–4.98)	
Tenotomy	No	14 (74%)	5 (26%)	1.00	0.85
	Yes	35 (71%)	14 (29%)	0.89 (0.27–2.95)	
Completed casting and fitted with brace	No	1 (20%)	4 (80%)	1.00	0.03*
	Yes	48 (76%)	15 (24%)	12.8 (1.3–123.5)	
Completed casting with Pirani score ≤ 1	No	11 (55%)	9 (45%)	1.00	0.05*
	Yes	38 (79%)	10 (21%)	3.11 (1.01–9.56)	
Brace use	< 2 years	17 (57%)	13 (43%)	1.00	0.02*
	≥ 2 years	32 (84%)	6 (16%)	4.08 (1.31–12.65)	

### Quality of life

There was a marked improvement in quality of life in all areas for those who complete casting compared to those who did not (Additional file 4). An ACT score 9–12 was associated with an increased quality of life (*p* = 0.002).

### Healthcare satisfaction

There was a tendency for parents whose children completed ≥2 years of bracing to be more satisfied (93, 95% CI: 88–99) with the information given to them in the clubfoot clinic than those who did not (85, 95% CI: 76–93) (Additional file 5) but this difference was not statistically significant.

## Discussion

A simple tool to assist non-specialist health workers to identify a good outcome after treatment with the Ponseti method from an outcome that needs further management is required. The four-question ACT tool was shown to have a high sensitivity and specificity in identifying children who need additional intervention.

There is no accepted gold standard to assess the results of clubfoot treatment. In the context of Africa, trained clubfoot therapists provide treatment. When children are seen during bracing, a decision on need for referral to an orthopaedic clinic is required. To assess whether the ACT tool could assist therapists in making that decision, the gold standard used was the agreement of two experienced physiotherapists after they had independently performed a full clinical assessment.

This study found that further intervention was indicated in 35% (24/68) of children and the success rate in those that completed casting and 2+ years of bracing was 82%. This indicates what can be achieved if there is good compliance with treatment and adequate follow-up. This is similar to high income settings where the probability of further intervention is reported as approximately 29% [21].

The ACT tool (one physical observation and 3 questions to the child’s carer) takes approximately five minutes to perform. There was excellent agreement in the results of the test between two different observers.

A score of 8 or less indicated that the child needed referral, whereas a score of 11 or 12 indicated the child had a good outcome. One child with a score of 9 and three children with a score of 10 were judged to need referral for more casting and one child with a score of 10 to need referral for surgical review. These cases included curvature of the lateral border of the foot and review for a tibialis anterior transfer. There were no cases that recorded low parent satisfaction or parent reported pain when the foot achieved plantigrade or more.

This was a cohort study with follow-up after first treatment of at least 3.5 years. Repeat phone calls facilitated attendance at the study clinic. The ACT tool was developed through an extensive Delphi process and literature review. The tool is simple and quick to administer and can be used by non-specialist health workers. It includes both physical observation and carer reported outcomes. The study protocol was pilot tested before use.

There were also study limitations. The study was undertaken in one clinic setting. Only 31.2% of the initial cohort were followed up. The cohort were potentially less severe at baseline, with mean initial Pirani scores of 4 or less. The results in those followed up (31.2%) are likely to be better than those for the cohort as they attended clinic appointments for longer and length of follow up is a predictor of good outcome. The tool is limited to one clinical examination, which restricts identification of pathology that is reliant on complex investigations. It is possible that results from the ACT tool may have influenced the decision to refer. Administering the tool first, but calculating the total score after the full clinical assessment, in addition to requiring agreement on the referral decision, should have reduced this potential for observer bias.

As non-specialist health workers regularly manage the treatment of clubfoot in low resource settings there is a need to provide appropriate tools to allow measurement and evaluation of their treatments. Further work is required to evaluate the ACT tool in other situations and with other cadres of clubfoot therapists. Also further exploration to differentiate children who score 9 or 10 with a good outcome from those who need referral is warranted; in particular the tool is not sensitive in identifying children who have a curvature in the front of the foot but who score high due to parent satisfaction, good footwear use and absence of pain. For example, the tool does not identify children who require tibialis anterior tendon transfer until they present with significant recurrent deformity. The score is excellent at providing a cross sectional assessment however further research is required to determine if it can detect earlier recurrence via the parent reported measures.

The use of the ACT score is to accurately inform and predict future management. It answers such questions as: (1) does the child need more treatment? (2) has the child been successfully treated? and (3) will the child’s quality of life be improved? It is suitable for use in children who are of walking age.

We recommend that the ACT tool is used on a yearly basis after completion of casting and commencement of bracing, or if the non-specialist health worker has concerns regarding the outcome of clubfoot treatment in a child of walking age. A score ≤ 8 predicts the need for further intervention. If a child scores 9 to 10, we recommend the clubfoot therapist identifies the primary reason and seeks a second opinion.

## Conclusion

This paper contributes to the data on the measurement of clubfoot treatment in low resource settings. The ACT tool includes a physical observation of the foot and parent reported outcome measures. A score ≤ 8 identifies children who need further intervention, and a score of 11 or 12 identifies children with a successful outcome. Further work is needed to distinguish the few children who have an ACT score of 9 or 10 and who require further treatment from those who have a successful outcome. There is an association between good outcome, high ACT score and higher quality of life.

## Additional files

Additional file 1: STARD Checklist for Reporting of Studies of Diagnostic Accuracy. (DOCX 21 kb)

Additional file 2: Results of ACT score and treatment required. (DOCX 12 kb)

Additional file 3: ACT score distribution. (DOCX 13 kb)

Additional file 4: Results of Quality of Life Questionnaire. (DOCX 13 kb)

Additional file 5: Results of Healthcare Satisfaction Questionnaire. (DOCX 15 kb)

## Abbreviations

ACT: assessing clubfoot treatment
CTEV: congenital talipes equinovarus
ROC: Receiver Operating Characteristic
