# Supplementary material for: Evaluation of a simple tool to assess the results of Ponseti treatment for use by clubfoot therapists: a diagnostic accuracy study
Source: J Foot Ankle Res. 2019 Mar 4;12:14. doi: 10.1186/s13047-019-0323-4 (PMC6399889; doi:10.1186/s13047-019-0323-4)
Supplement: Supplementary file 2 — Results of ACT score and treatment required. (DOCX 12 kb) [file 13047_2019_323_MOESM2_ESM.docx]

**Additional File 2: Results of ACT score and treatment required**

| ACT score | Number of children with no intervention required | Number of children who required re-casting | Number of children who required surgical review |
| --- | --- | --- | --- |
| 0 |  |  | 1 |
| 4 |  |  | 3 |
| 5 |  |  | 1 |
| 6 |  | 1 | 4 |
| 7 |  | 1 | 3 |
| 8 |  | 1 | 4 |
| 9 | 5 | 1 |  |
| 10 | 3 | 3 | 1 |
| 11 | 12 |  |  |
| 12 | 24 |  |  |
