# Supplementary material for: Evaluation of a simple tool to assess the results of Ponseti treatment for use by clubfoot therapists: a diagnostic accuracy study
Source: J Foot Ankle Res. 2019 Mar 4;12:14. doi: 10.1186/s13047-019-0323-4 (PMC6399889; doi:10.1186/s13047-019-0323-4)
Supplement: Supplementary file 3 — ACT score distribution. (DOCX 13 kb) [file 13047_2019_323_MOESM3_ESM.docx]

**Additional File 3: ACT score distribution**

|  | | Score 3 | Score 2 | Score 1 | Score 0 |
| --- | --- | --- | --- | --- | --- |
|  |  | N (%) | N (%) | N (%) | N (%) |
| Total Cohort followed up (n=68) | Foot is plantigrade | 33 (49%) | 22 (32%) | 7 (10%) | 6 (9%) |
|  | Complain of pain | 44 (65%) | 18 (27%) | 5 (7%) | 1 (1%) |
|  | Wears shoe of choice | 47 (69%) | 13 (19%) | 6 (9%) | 2 (3%) |
|  | Satisfied with foot | 42 (62%) | 14 (21%) | 9 (13%) | 3 (4%) |
| Completed casting (n=63) | Foot is plantigrade | 31 (49%) | 22 (35%) | 7 (11%) | 3 (5%) |
|  | Complain of pain | 43 (68%) | 15 (24%) | 5 (8%) | 0 (0%) |
|  | Wears shoe of choice | 45 (71%) | 12 (19%) | 6 (10%) | 0 (0%) |
|  | Satisfied with foot | 41 (65%) | 12 (19%) | 9 (14%) | 1 (2%) |
| Completed ≥2 years bracing (n=38) | Foot is plantigrade | 22 (58%) | 15 (39%) | 1 (3%) | 0 (0%) |
|  | Complain of pain | 27 (71%) | 9 (24%) | 2 (5%) | 0 (0%) |
|  | Wears shoe of choice | 27 (71%) | 8 (21%) | 3 (8%) | 0 (0%) |
|  | Satisfied with foot | 28 (74%) | 6 (16%) | 4 (10%) | 0 (0%) |
