# Supplementary material for: Evaluation of a simple tool to assess the results of Ponseti treatment for use by clubfoot therapists: a diagnostic accuracy study
Source: J Foot Ankle Res. 2019 Mar 4;12:14. doi: 10.1186/s13047-019-0323-4 (PMC6399889; doi:10.1186/s13047-019-0323-4)
Supplement: Supplementary file 4 — Results of Quality of Life Questionnaire. (DOCX 13 kb) [file 13047_2019_323_MOESM4_ESM.docx]

**Additional File 4: Results of Quality of Life Questionnaire** (Higher % = higher perceived quality of life)

| Quality of life Dimension** | Did not complete casting | Completed casting, but <2 years bracing | Completed casting, and 2+ years bracing | Total cohort followed up** |
| --- | --- | --- | --- | --- |
|  | N = 6 | N = 24 | N = 38 | N= 62 |
|  | Mean (95% CI) | Mean (95% CI) | Mean (95% CI) | Mean (95% CI) |
| Physical functioning | 68  (27 – 100) | 88  (81 – 96) | 96  (93 – 99) | 93  (89 – 96) |
| Emotional functioning | 69  (54 – 82) | 84  (76 – 91) | 89  (85 – 94) | 87  (83 – 91) |
| Social functioning | 75  (41 – 100) | 85  (78 – 93) | 95  (93 – 98) | 91  (87 – 95) |
| School functioning | 65  (15 – 100) | 81  (72 – 91) | 94  (89 - 99) | 88  (83 – 93) |
| Psychosocial health | 72  (47 – 96) | 83  (77 – 91) | 93  (90 – 96) | 89  (86 – 93) |
| **Total score** | **69**  **(31 – 100)** | **86**  **(80 – 92)** | **94**  **(92 – 97)** | **91**  **(87 – 94)** |

**data missing from 6 children
