# Supplementary material for: Evaluation of a simple tool to assess the results of Ponseti treatment for use by clubfoot therapists: a diagnostic accuracy study
Source: J Foot Ankle Res. 2019 Mar 4;12:14. doi: 10.1186/s13047-019-0323-4 (PMC6399889; doi:10.1186/s13047-019-0323-4)
Supplement: Supplementary file 5 — Results of Healthcare Satisfaction Questionnaire. (DOCX 15 kb) [file 13047_2019_323_MOESM5_ESM.docx]

**Additional File 5: Results of Healthcare Satisfaction Questionnaire** (Higher % = higher satisfaction)

| Healthcare satisfaction Dimension** | Cohort Mean % (95%CI)  n=64 | Complete casting Mean % (95%CI)  n=60 | Did not complete casting mean % (95%CI) n=4 | P-value | Pirani ≤1  % (95%CI)  (n=46) | Pirani ≥1  % (95%CI)  (n=18) | P-value | Children completing ≥2 years of bracing (n=37) | Children who do not complete 2 years (n=27) | P-value |
| --- | --- | --- | --- | --- | --- | --- | --- | --- | --- | --- |
| Information | 83  (77 – 88) | 84  (78 – 90) | 70  (0 – 100) | 0.49 | 82  (75 – 88) | 85  (72 – 97) | 0.56 | 88  (83 – 96) | 75  (64 – 86) | **0.02*** |
| Inclusion of family | 82  (76 – 87) | 82  (76- 87) | 77  (21 – 100) | 0.25 | 81  (75 – 87) | 84  (70 – 97) | 0.50 | 83  (76 – 90) | 80  (71 – 90) | 0.63 |
| Communication | 83  (77- 88) | 83  (78 – 88) | 74  (0 – 100) | 0.64 | 82  (76 – 87) | 85  (69 – 99) | 0.53 | 83  (76-90) | 82  (72 – 92) | 0.87 |
| Technical skills | 88  (83 – 92) | 88  (83 – 92) | 88  (48 – 100) | 0.42 | 87  (82 – 92) | 90  (79 – 99) | 0.45 | 87  (80 – 93) | 89  (82 – 95) | 0.67 |
| Emotional needs | 74  (66 – 82) | 74  (66 – 82) | 74  (0 – 100) | 0.98 | 75  (66 – 83) | 71  (50 – 92) | 0.81 | 72  (61 – 83) | 76  (64 – 88) | 0.61 |
| Overall satisfaction | 90  (85 – 95) | 91  (86 – 95) | 74  (4 – 100) | 0.98 | 91  (86 – 95) | 87  (73 – 99) | 0.59 | 93  (88 – 99) | 85  (76 – 93) | **0.07*** |
| Total score | 83  (78 – 88) | 83  (79 – 88) | 76  (10 – 100) | 0.18 | 83  (78 – 88) | 82  (69 – 96) | 0.82 | 84  (78 – 90) | 81  (73 – 90) | 0.54 |

**data from 4 children missing
